# Should I stay or should I go again: Multiple switching between fee‐for‐service Medicare and Medicare advantage among older beneficiaries

**DOI:** 10.1111/1475-6773.14398

**Published:** 2024-10-17

**Authors:** Geoffrey J. Hoffman, Yang Amy Jiao, Zhaohui Fan, H. Myra Kim, Lillian Min, Donovan Maust

**Affiliations:** ^1^ Department of Systems, Populations, and Leadership University of Michigan School of Nursing Ann Arbor Michigan USA; ^2^ Institute for Healthcare Policy and Innovation University of Michigan Ann Arbor Michigan USA; ^3^ Department of Health Management and Policy University of Michigan School of Public Health Ann Arbor Michigan USA; ^4^ Center for Healthcare Outcomes & Policy University of Michigan Ann Arbor Michigan USA; ^5^ Consulting for Statistics, Computing & Analytics Research (CSCAR) University of Michigan Ann Arbor Michigan USA; ^6^ Division of Geriatric and Palliative Medicine, Department of Internal Medicine University of Michigan Medical School Ann Arbor Michigan USA; ^7^ Geriatric Research Education and Clinical Center Ann Arbor VA Healthcare System Ann Arbor Michigan USA; ^8^ Department of Psychiatry University of Michigan Ann Arbor Michigan USA; ^9^ Center for Clinical Management Research Virginia Ann Arbor Healthcare System Ann Arbor Virginia USA

**Keywords:** Health‐care financing/insurance, health economics, managed care organizations, Medicare

## Abstract

**Objective:**

To evaluate whether having previously disenrolled from Medicare Advantage (MA) is associated with lower hazards of future MA enrollment.

**Data Sources and Study Setting:**

Secondary data from Medicare.

**Study Design:**

We examined beneficiaries with baseline FFS enrollment from 2017–2019 using a 20% sample of Medicare claims. Cox proportional hazard models were used to examine the association of prior MA enrollment (in the three years prior to baseline FFS enrollment) with MA re‐enrollment, and whether this association is modified by Alzheimer's Disease and Related Dementias (ADRD), prior nursing home use, chronic illness, dual eligible status, and availability of MA plans and quality.

**Data Collection:**

Not applicable.

**Principal Findings:**

Overall, 3.3% of beneficiaries switched to MA annually. Of those with prior MA enrollment, MA switching percentages were 9.0%, 4.6%, and 6.8% for those whose most recent MA enrollments were 1, 2, and 3 years prior to their baseline FFS year. Comparatively, the switching percentages was 3.2% for those with no prior MA enrollment. The hazards of switching to MA were 2.73 (*p* < 0.001), 1.29 (*p* < 0.001), and 1.97 (*p* < 0.001) times greater than remaining in FFS for beneficiaries whose most recent MA enrollments were one, two, and three years prior to their baseline FFS year. Hazards of switching were generally similar between those with and without ADRD, stratified by recency in prior MA experience, except those with dual eligibility. Among those with ADRD, switching hazards were greatest for 3 years prior MA enrollees in counties with the fewest available (HR: 3.84, *p* < 0.001) and lowest‐rated plans (HR: 4.02, *p* < 0.001).

**Conclusions:**

Recency of switching from MA to FFS was the strongest predictor of a FFS‐to‐MA switch, identifying a population of beneficiaries who multiply switch regardless of health status or MA access. Future health policy considerations should more closely examine the vulnerabilities and long‐term outcomes of this population.

**What is known on this topic**

- Four to five percent of Medicare beneficiaries switch out of MA for FFS annually, and roughly half disenroll from MA contracts after five years.
- Exit rates out of MA for FFS are greater among high‐risk populations, who may be dissatisfied with higher‐than‐expected out‐of‐pocket costs, restricted provider networks, or low‐quality post‐acute care.

**What this study adds**

- Beneficiaries who had earlier exited MA had substantially higher hazards of switching back to MA compared to FFS beneficiaries who had not previously enrolled in MA.
- These trends were observed across high‐risk populations known for high MA exit rates and were more pronounced in counties with fewer and lower‐rated MA plans.
- Dissatisfaction with MA driving MA exits may not be lasting. Beneficiaries may temporarily be using FFS for enhanced coverage during health crises.

## 1 INTRODUCTION

Medicare Advantage (MA), which offers a range of private health plans as alternatives to traditional fee‐for‐service (FFS) Medicare, has experienced rapid recent growth and currently enrolls nearly half of all Medicare beneficiaries.^1^ Compared to FFS, MA generally provides similar or higher‐quality care and care management, and in many cases offers relatively lower premiums and broader benefits than FFS.^2^, ^3^

Despite the popularity of MA, four to five percent of Medicare beneficiaries switch out of MA for FFS annually, and roughly half disenroll from MA contracts after five years.^4^, ^5^ Concerningly, exit rates out of MA for FFS are greater among high‐risk populations,^4^, ^6^, ^7^, ^8^ who may be dissatisfied with higher‐than‐expected out‐of‐pocket costs, restricted provider networks, or low‐quality post‐acute care.^2^, ^9^ Annual exit rates from MA to FFS are 2%–5%, 10% for those with cognitive impairment, and as high as 20% for higher‐risk individuals requiring post‐acute care services.^4^, ^6^, ^7^

However, prior investigations have primarily looked at point‐in‐time enrollment changes. Yet, rather than lasting dissatisfaction with care quality, MA exits may represent efficient sorting of consumers, as beneficiaries pursue higher value care over time, depending on their health needs. For instance, higher‐risk beneficiaries may opt for FFS coverage when care needs are high—given no restriction networks or utilization review in FFS—but then later return to MA. If so, interpreting MA exits as signals of poor quality may misunderstand consumer behavior, and fail to understand longer‐term trends in MA and FFS use that may require policy attention.

In this study, we build on existing work by evaluating the likelihood of returning to MA after an earlier exit, including for high‐risk patients such as those with dementia. We then ascertain if these switching patterns vary according to patient risk (nursing home use, chronic illness, dual eligibility status) or market factors (the number and quality of MA plans available in each county), to better understand how MA exits reflect consumer behaviors and insurance alternatives. Our objective is to offer policymakers insights into characteristics of patients who recently switched out of MA to FFS to see whether they would be more likely than other FFS beneficiaries to stay in FFS (which would suggest dissatisfaction with earlier MA experience) or to again enroll in MA (which would suggest less dissatisfaction with earlier MA experience or dissatisfaction with FFS, among other factors driving enrollment choices).

## 2 METHODS

The University of Michigan Institutional Review Board approved this study and waived the informed consent requirement because the study involved national claim databases and data were de‐identified. We followed the Strengthening the Reporting of Observational Studies in Epidemiology (STROBE) reporting guidelines.

### 2.1 Data, cohort, and measures

We used Medicare claims and the Medicare Master Beneficiary Summary File (MBSF) to obtain a 20% national random sample of FFS Medicare beneficiaries (i.e., those with Medicare Parts A and B and no Part C coverage) for the period 2014 to 2020. We only included individuals residing in one of the 50 US states or the District of Columbia and those with non‐missing ZIP‐code information, resulting in 11,016,947 observations for 3,993,024 beneficiaries. The MBSF was used to identify monthly enrollment. We identified a cohort of Medicare beneficiaries who were enrolled in FFS for at least one complete calendar year during the 2017–2019 period and who had at least one subsequent year of follow‐up Medicare enrollment. Dually eligible beneficiaries were excluded from our main analyses because they are able to enroll in MA or FFS at multiple points during the year without penalty or restrictions.

### 2.2 Primary exposure variable

For each beneficiary in the cohort, we defined three distinct evaluation periods (see Figure S1 in the Appendix): (1) the “baseline FFS” year, or the beneficiary's first full year of FFS enrollment during the 2017–2019 period; (2) the “follow‐up,” a period of up to three years following a beneficiary's baseline FFS year; and (3) the three‐year “lookback” period, which captures a beneficiary's most recent MA enrollment, if any, prior to the baseline FFS year. Data were pooled such that the baseline FFS year could be any of 2017, 2018, or 2019; we examined all data together from this final, pooled cohort. To ensure that all beneficiaries had the full three years of MA lookback, we only included beneficiaries who were at least 68 years old during their baseline FFS year.

### 2.3 Covariates

Age, gender, and race were obtained at each beneficiary's baseline FFS year. Race was measured using the MBSF's enrollment database measure. We examine beneficiary‐level race and other, area‐level measures of race/ethnicity (discussed below) in order to capture potential structural determinants of health and consumer preference. The 20% of claims files contain diagnoses and billing codes that can be used to identify ADRD, comorbidities, and health‐care utilization information. We identified individuals with ADRD in the baseline year using the Bynum 1‐Year Standard Method, which has strong properties for identifying ADRD populations.^10^ This algorithm uses diagnoses from all‐claims settings (including hospital, skilled nursing facility, and home health care) to identify beneficiaries with dementia. It modifies the commonly used CMS Chronic Conditions Warehouse (CCW) algorithm by adding several diagnoses (e.g., Lewy‐Body dementia, other cerebral degeneration) and by examining one year rather than three years of claims. When validated against a reference standard from the Health and Retirement Study, the Bynum algorithm had lower ADRD prevalence than the CCW (5.8% vs. 11.1%), but better properties (specificity: 98.0% vs. 92.3%; positive predictive value: 80.3% vs. 53.8%).

Time‐varying covariates were annually measured in the baseline and subsequent years until a switch to MA or up to the third year after the baseline year for those beneficiaries without a switch from FFS to MA. These included numbers of comorbidities, and lagged numbers of inpatient stays, skilled nursing admissions, and home health agency visits (measured one year prior), as previous studies have shown that people with higher health‐care utilization are more likely to switch between FFS and MA.^8^ Year‐specific county‐level characteristics were obtained from the Agency on Healthcare Research and Quality's Social Determinants of Health (SDOH) database. These included ZIP‐code level median household income and the relative percentages of Black, Hispanic, and Asian residents in the beneficiary's county of residence, and the county‐level supply of specialists and primary care physicians per 100,000 residents. We used the American Hospital Association's annual survey to obtain annual county‐level hospital bed counts and proportions of hospitals that are teaching hospitals, to adjust for local health‐care resources and practices that could influence MA enrollment choice. Finally, counties' annual number of MA plans and MA penetration rate (percent of older adults enrolled in MA) were included, as these have been shown to strongly influence the likelihood of MA plan offerings, MA uptake, and both FFS and MA spending.^11^, ^12^, ^13^

### 2.4 Statistical analysis

We first described the main study sample overall (not including dual eligibles), by subpopulation, and by whether beneficiaries had prior MA enrollment. We also described enrollment for beneficiaries enrolled in MA both prior to and after FFS enrollment. Specifically, we assessed the percentages of these beneficiaries enrolled in specific plan types (e.g., Health Maintenance Organization [HMO], local or regional Preferred Provider Organization [PPO]) and plans of varying quality (i.e., high [4–5 stars], medium [3–3.5 stars], or low [2–2.5 stars] ratings). We computed the annual switching rate per 100 beneficiaries.

Our outcome of interest was MA plan enrollment during beneficiaries' follow‐up period. We used Cox Proportional Hazards models to examine the time to a first switch to MA during the three years after the baseline FFS year. Time was censored if death occurred before 3 years (with approximately 9.2% of all beneficiaries died, including 8.8% who were censored due to death).

To evaluate the impact of prior MA enrollment, the models included three mutually exclusive prior MA enrollment indicators representing whether, during the MA lookback period, beneficiaries' most recent MA enrollment occurred one, two, or three years prior to their baseline FFS year. Beneficiaries without any MA enrollment during the MA lookback period were the reference group. An indicator for ADRD and interaction terms between each of the three prior MA experience indicators and the ADRD indicator were included to assess whether the impact of prior MA enrollment on MA switching differed according to ADRD status.

Using the main sample (not including dual eligibles), we also stratified the analysis according to several beneficiary and area characteristics. First, we separately looked at those who had a skilled nursing facility admission (identified using MedPAR claims) or presence of one or more chronic illnesses (defined using the Charlson comorbidity index) during the baseline FFS year to explore if the relationship between prior MA enrollment and switching back to MA differed in beneficiaries with known high risk of MA exits.^4^ We also separately stratified models by each of a county's availability of MA plans in a county and average MA plan star ratings to see how insurance alternatives factor into MA exit and return behaviors.

Finally, in a sensitivity analysis, we separately identified and analyzed risks of switching for 562,764 dually eligible beneficiaries. This population was identified as those beneficiaries with at least 3 months of dual eligibility (defined as any state buy‐in for Medicare Part A or B) during the baseline FFS year.

All models were adjusted for baseline FFS year age, gender, and race, beneficiary‐level time‐varying characteristics, year‐specific contextual factors (county‐level sociodemographic, clinician supply, and MA enrollment characteristics), as described above, and county fixed effects. Covariate missingness was limited (around 0.0%–0.2%) (see Table S1).

We calculated hazard ratios using the coefficients from the Cox model. All analyses were conducted between Jan 2023 and Dec 2023 using SAS, 9.4 (SAS Institute).

## 3 RESULTS

### 3.1 Unadjusted results

We identified a total of 3,993,024 beneficiaries with baseline FFS enrollment from 2017–2019. Most beneficiaries were White (88.4%, versus 6.1% Black and 0.7% Hispanic), 53.9% were female, and 7.8% had ADRD (Table S2).

Among all beneficiaries, 3.7% (146,079 of 3,993,024) had MA enrollment in the prior three years (Table S2). For beneficiaries with compared to without prior MA enrollment, smaller percentages were White (86.7% vs. 88.5%) but more had ADRD (8.2% vs. 7.8%). Beneficiaries with compared to without prior MA enrollment were from counties with more MA plans (40.0 vs. 38.9) and higher MA penetration rates (32.8% vs. 30.3%).

Overall, the annual switching rate (from FFS to MA) was 3.3 per 100 Medicare beneficiaries (Table 1). Among those with no prior MA enrollment, the rate was 3.2, while among those with prior MA enrollment it was 6.5 overall, and 9.0, 4.6, and 6.8 per 100 beneficiaries among those whose most recent MA enrollments were one, two, and three years prior to their baseline FFS year, respectively. The annual switching rate was 3.0 for FFS beneficiaries with ADRD. This included rates of 2.8 for those who had no prior MA enrollments, 6.4 for those with any prior MA enrollment, and 8.3, 4.0, and 7.4 per 100 beneficiaries for those whose most recent MA enrollments were one, two, and three years prior to their baseline FFS year, respectively. For dual eligibles, there was a 7.0 overall annual switching rate overall and 12.0 switching rate among those with any prior MA enrollment.

**TABLE 1:** Unadjusted annual rate of switching from FFS to MA.

		Overall *N* (%)	ADRD *N* (%)	NH *N* (%)	Chronic illness *N* (%)	Dual eligible *N* (%)
Overall	*N*	3,993,024	312,142	140,417	1,716,118	595,580
	Years	11,016,947	741,830	329,192	4,605,863	1,485,993
	Rate	3.3	3.0	2.7	3.2	7.0
No prior MA	*N*	3,846,945	300,156	134,666	1,648,289	542,750
	Years	10,626,259	714,329	315,957	4,428,040	1,362,411
	Rate	3.2	2.8	2.6	3.0	6.6
Prior MA						
Any year	*N*	146,079	11,986	5751	67,829	52,830
	Years	390,688	27,501	13,235	177,823	123,582
	Rate	6.5	6.4	6.1	6.1	12.0
Year −1	*N*	37,171	3075	1575	16,357	19,741
	Years	94,970	6588	3517	41,230	44,002
	Rate	9.0	8.3	8.0	8.1	15.0
Year −2	*N*	53,410	4016	2054	25,148	16,938
	Years	146,132	9435	4805	67,221	40,269
	Rate	4.6	4.0	3.4	4.1	10.7
Year −3	*N*	55,498	4895	2122	26,324	16,151
	Years	149,586	11,478	4913	69,372	39,311
	Rate	6.8	7.4	7.3	6.8	10.1

*Note*: *N* represents the number of beneficiaries and *Years* represents the number of beneficiary years in each population. The rate indicates the rate of switching from FFS to MA in a given year, per 100 beneficiaries. Information for dual eligibles is presented in the “Dual Eligible” column. However, dual eligibles were *not* included in any of the values for other populations (i.e., overall, ADRD, nursing home users, and those with chronic illness).

Abbreviations: FFS, fee‐for‐service; MA, Medicare Advantage; NH, nursing home. Years −1, −2, and −3 represent most recent MA enrollment in 1, 2, and 3 years prior to the FFS baseline year.

### 3.2 Changes in MA plan types among MA re‐enrollees

We explored differences in MA plan enrollment among MA re‐enrollees. Specifically, we compared changes in plan type and quality at re‐enrollment (i.e., for beneficiaries who re‐enrolled in MA in the follow‐up period) compared to at a prior MA enrollment (i.e., for beneficiaries enrolled in MA before their baseline FFS enrollment). We observed slight shifts in plan type and quality, with more beneficiaries re‐enrolling in local PPOs and in higher‐quality plans, when compared to prior enrollments. Specifically, at re‐enrollment compared to prior enrollment, fewer beneficiaries enrolled in HMOs (52.1% before FFS enrollment vs. 59.5% after FFS enrollment) and in regional PPOs (5.4% vs. 9.1%), but more enrolled in local PPOs (37.7% vs. 30.7%) (Figure S2). Additionally, at re‐enrollment compared to prior enrollment, more beneficiaries enrolled in top‐rated plans (ratings of 4–5 stars) (73.4% vs. 64.1%) and fewer were in plans with moderate (ratings of 3–3.5 stars) and lower quality (ratings of 2–2.5 stars) (26.2% vs. 33.3% for moderate quality plans and 0.4% vs. 2.8% for low‐quality plans) (Figure S3).

### 3.3 Adjusted results

#### 3.3.1 Overall

**FIGURE 1:**
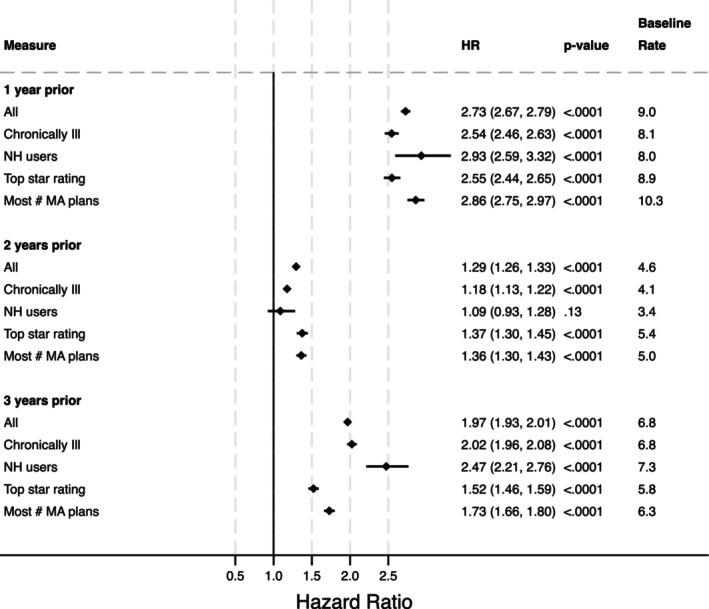
Differences in switching for all non‐dual eligibles (“All Individuals”) and among dual eligibles only. Authors' analysis of data from the Medicare Master Beneficiary Summary File (MBSF) linked to Medicare claims. Provider and MA plan characteristics are from the Agency on Healthcare Research and Quality's Social Determinants of Health (SDOH) database and Medicare Advantage/Part D Contract and Enrollment Data. Hazard ratios are calculated from the Cox model coefficients. *p*‐values are for the contrasts between a given insurance category and the omitted reference, i.e., for those with MA enrollment 1 year prior compared to those with no prior MA enrollment.

In the main sample not including dual eligibles, compared to those with no prior MA enrollment in the past three years, beneficiaries whose most recent MA enrollment was one, two, and three years prior to their FFS baseline had 2.73 (HR: 2.73; 95% CI: 2.67, 2.79, *p* < 0.001) 1.29 (95% CI: 1.26, 1.33, *p* < 0.001), and 1.97 (95% CI: 1.93, 2.01, *p* < 0.001) times higher hazard of switching to MA, respectively (Figure 1).

#### 3.3.2 By ADRD status

**FIGURE 2:**
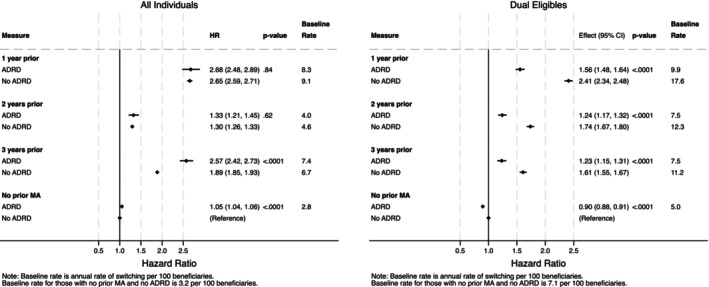
Differences in switching for all non‐dual eligibles (“All Individuals”) and among dual eligibles only, by ADRD status. Authors' analysis of data from the Medicare Master Beneficiary Summary File (MBSF) linked to Medicare claims. Provider and MA plan characteristics are from the Agency on Healthcare Research and Quality's Social Determinants of Health (SDOH) database and Medicare Advantage/Part D Contract and Enrollment Data. Hazard ratios are calculated from the Cox model coefficients. Non‐ADRD beneficiaries with no prior MA enrollment are the reference group. The *p*‐values show the significance level comparing ADRD and non‐ADRD populations. *p*‐values are for the contrasts between ADRD and non‐ADRD populations, i.e., the risk differences in FFS‐to‐MA switching for those with versus without ADRD among those with MA enrollment 1 year prior compared to those without ADRD who had no prior MA experience. ADRD, Alzheimer's Disease and Related Dementias; HR, hazard ratio; MA, Medicare Advantage.

Compared with beneficiaries without prior MA enrollment, beneficiaries with MA enrollment in the prior 3 years had greater rates of switching to MA whether they had ADRD or not. For example, compared to those with no prior MA enrollment and no ADRD, beneficiaries with (HR: 2.68; 95% CI: 2.48, 2.89, *p* < 0.001) and without ADRD (HR: 2.65; 95% CI: 2.59, 2.71, *p* < 0.001) who were enrolled in MA one year prior had similar hazards of switching to MA (*p* = 0.84 for difference between those with and without ADRD) (Figure 2 and Table S3). Hazards of switching were also similar in magnitude between those with versus without ADRD among those with MA experience that was two years prior (*p* = 0.62). However, beneficiaries with (HR: 2.57; 95% CI: 2.42, 2.73, *p* < 0.001) versus without ADRD (HR: 1.89; 95% CI: 1.85, 1.93, *p* < 0.001) had substantially higher rates of enrolling in MA (*p* < 0.001 for difference between those with and without ADRD) among those with MA enrollment three years prior compared to those with no prior MA enrollment.

#### 3.3.3 Sub‐group analyses by beneficiary characteristics

**FIGURE 3:**
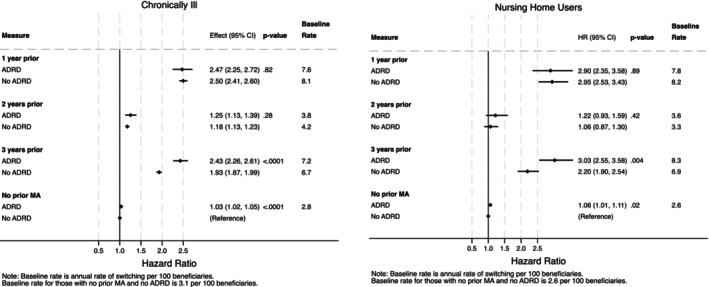
Differences in switching according to chronic illness status and nursing home use, by ADRD status. Authors' analysis of data from the Medicare Master Beneficiary Summary File (MBSF) linked to Medicare claims. Provider and MA plan characteristics are from the Agency on Healthcare Research and Quality's Social Determinants of Health (SDOH) database and Medicare Advantage/Part D Contract and Enrollment Data. Hazard ratios are calculated from the Cox model coefficients. Non‐ADRD beneficiaries with no prior MA enrollment are the reference group. The *p*‐values show the significance level comparing ADRD and non‐ADRD populations. Chronically ill beneficiaries are those with one or more comorbidity, identified using the Charlson comorbidity index. *p*‐values are for the contrasts between ADRD and non‐ADRD populations, i.e., the risk differences in FFS‐to‐MA switching for those with versus without ADRD among those with MA enrollment 1 year prior compared to those without ADRD who had no prior MA experience. ADRD, Alzheimer's Disease and Related Dementias; HR, hazard ratio; MA, Medicare Advantage.

Compared to the main analyses, results were similar when examining sub‐groups (not including dual eligibles) considered at high risk for exiting MA. For instance, among those with at least one chronic illness (defined using the Charlson comorbidity index), the relative hazards of switching from FFS to MA for those with and without ADRD respectively were approximately two‐and‐a‐half times as high for those whose most recent MA experiences were respectively one year (HR: 2.47; 95% CI: 2.25, 2.72, *p* < 0.001 for those with ADRD and HR: 2.50; 95% CI: 2.41, 2.60, *p* < 0.001 for those without ADRD; *p* = 0.82 for difference between those with and without ADRD) and three years (HR: 2.43; 95% CI: 2.26, 2.61, *p* < 0.001 for those with ADRD and 1.93; 95% CI: 1.87, 1.99, *p* < 0.001 for those without ADRD; *p* < 0.001 for difference) prior to their baseline FFS year compared to those with no prior MA enrollment (Figure 3 and Table S4). Additional results can be found for those with prior nursing home use in Figure 3 and Table S5. ADRD status was associated with differential likelihood of enrollment in MA (with greater enrollment likelihood for those with ADRD) in each of the chronically ill and nursing home user sub‐groups, among those with prior MA enrollment three years earlier.

#### 3.3.4 Sub‐group analyses by area characteristics

**FIGURE 4:**
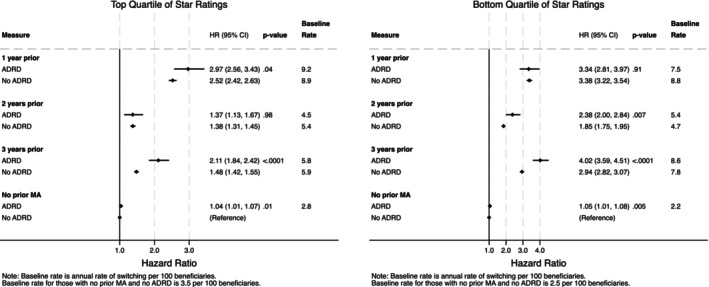
Differences in switching according to average area MA plan star ratings, by ADRD status. Authors' analysis of data from the Medicare Master Beneficiary Summary File (MBSF) linked to Medicare claims. Provider and MA plan characteristics are from the Agency on Healthcare Research and Quality's Social Determinants of Health (SDOH) database and Medicare Advantage/Part D Contract and Enrollment Data. Hazard ratios are calculated from the Cox model coefficients. Non‐ADRD beneficiaries with no prior MA enrollment are the reference group. The *p*‐values show the significance level comparing ADRD and non‐ADRD populations. *p*‐values are for the contrasts between ADRD and non‐ADRD populations, i.e., the risk differences in FFS‐to‐MA switching for those with versus without ADRD among those with MA enrollment 1 year prior compared to those without ADRD who had no prior MA experience. ADRD, Alzheimer's Disease and Related Dementias; HR, hazard ratio; MA, Medicare Advantage.

In counties with the fewest available and lowest‐rated MA plans to choose from and counties with high levels of prior FFS‐to‐MA switching, beneficiaries with ADRD and MA in 3 years prior had approximately four times the hazard of switching to MA than beneficiaries with no prior MA and without ADRD: specifically, 4.02 times higher (95% CI: 3.59, 4.51, *p* < 0.001) for counties with the lowest average star ratings (Figure 4) and 3.84 times higher hazards (95% CI: 3.41, 4.33, *p* < 0.001) for counties with the fewest numbers of plans (Figure S4).

#### 3.3.5 Dual eligibles

For the dual eligible population, unlike in the main population that did not include dual eligibles, ADRD populations had consistently lower hazards of enrolling in MA compared to non‐ADRD populations, both for those with and without prior MA enrollment and regardless of ADRD status (Figure 2 and Table S6).

## 4 DISCUSSION

In this study, we observed high FFS‐to‐MA insurance switching rates for older Medicare beneficiaries with and without ADRD, particularly for beneficiaries who had earlier exited MA. Annual switching rates were nearly double for those with versus without prior enrollment in MA. These trends were observed across a number of high‐risk populations known for high MA exit rates and was more pronounced in counties with fewer and lower‐rated MA plans. Collectively, this suggests that MA users may shop around substantially, which could reflect efforts to improve health‐care coverage over time, under differing health‐care needs.

Prior research suggests that initial insurance coverage decisions for Medicare beneficiaries are generally long‐lasting, or “sticky,” and that MA beneficiaries in particular are generally happy with their plans.^14^ However, a growing literature has identified churn within and outside MA,^5^ and that MA beneficiaries consider insurance enrollment changes when their health declines.^14^ This has been interpreted as signaling dissatisfaction with MA plans that may be unable to provide high‐quality care coordination for patients with complex care needs.^4^, ^7^, ^8^, ^9^

For instance, MA‐to‐FFS exit rates of 9% and 17% have been observed, compared to FFS‐to‐MA exit rates of just 4% and 3% for short‐ and long‐term nursing home users.^4^

However, our work indicates that, for some beneficiaries, MA exits are impermanent. That is, prior (and, particularly, more recent) experience with MA is a driver of future Medicare enrollment decisions. After experiencing both MA and FFS, some older Medicare beneficiaries—including those with acute care needs and post‐acute utilization—choose to re‐enroll in MA. Because the annual switching rate was only 3.3% (and 6.5% for those with prior MA enrollment), our findings still indicate a preponderance of enrollees exiting MA. Nonetheless, bouncing between MA contracts^5^ or out of and then back into MA may be more common than previously believed, with nearly 150,000 annual switches back to MA among those who had previously left the private Medicare insurance program. For context, concerns about MA disenrollments and potential quality deficits reflect MA exit rates of 10% or higher,^4^, ^6^ or roughly equivalent rates as those of re‐entry seen among some sub‐groups in this study. Among MA re‐entrants, therefore, reasons for exiting MA either may not be lasting or simply are not the drivers for disenrollment that have been implied by prior work using claims data (e.g., poor‐quality care) showing exits after high‐risk and high‐cost periods for MA enrollees.

Our finding that MA re‐enrollments were somewhat common among high‐risk beneficiaries also updates understandings from studies finding that cognitive impairment depresses enrollment or signals poor‐quality care.^12^ Ankuda et al.^6^ found that two‐and‐a‐half times as many beneficiaries with functional disability (a subpopulation that commonly includes individuals with cognitive impairment) switched out of MA and into FFS compared to into MA from FFS; the authors concluded that high disenrollment rates were potentially due to poor post‐acute care quality or beneficiaries' low profitability for MA plans given that MA risk‐adjustment does not account for functional measures. However, we identified higher enrollment rates in MA for those with versus without impairment among those with no prior MA enrollment, and similar re‐enrollment rates for those with prior MA exposure. Differences in MA enrollment by ADRD status were only observed among the dually eligible population, who can enroll in MA Special Needs Plans.^15^

One takeaway is therefore that earlier MA exits, or any dissatisfaction driving those exits, are not always long‐term deterrents to future MA use. It may be that consumers switch to less restrictive (in terms of provider network) FFS coverage after a health event, then switch back to relatively more comprehensive (in terms of benefits) if more restrictive insurance coverage under an MA plan later on, perhaps after stabilization or updates to patient perceptions of future need.^14^, ^16^ FFS costs may also be highly salient in driving decisions to re‐enter MA. This could reflect the inability of higher‐risk beneficiaries leaving MA for FFS to obtain supplemental (Medigap) coverage due to underwriting, making FFS coverage unaffordable without cost‐sharing assistance.^17^, ^18^ While our findings do not support this hypothesis, we were not able to directly evaluate Medigap enrollment and instead examined states in which such coverage is less likely.

Prior research also underlines the salience of plan premiums and other costs in coverage decisions.^8^, ^14^, ^16^, ^19^ For instance, of MA beneficiaries who took Medicare's Disenrollment Reasons Survey, approximately one‐quarter reported an increase in their plan premium as a factor for disenrollment, while more than four in ten indicated that they had found a plan that costs less.^16^ Moreover, coverage costs are substantially lower for dual eligibles, among whom observed risks of MA re‐entry were substantially lower in our study.

On the other hand, MA exits and re‐entry may not uniquely reflect dissatisfaction with quality or costs in MA or FFS, but instead other consumer shopping behaviors. For instance, recent work suggests that other factors, such as individuals following their spouse or partner's choice, may also be driving enrollment behaviors.^20^ Similarly, our results are consistent with consumers leveraging Medicare coverage options inside and outside the MA marketplace to improve their health‐care coverage over time, by identifying more generous (i.e., PPO plans with broader provider networks) and higher‐quality (i.e., higher star‐rated) MA plan options.

Finally, MA may also be effective at attracting lower‐risk and more “favorable” consumers,^21^ including those who have previously disenrolled. This may be due to consumer preferences for MA's generally lower premiums,^14^ even beyond satisfaction with care experiences.

### 4.1 Limitations

While this study has a number of strengths, including its novel examination of multiple switching over the longer‐term, it has several limitations worth noting. Other factors we did not examine, including out‐of‐pocket costs, could be unseen drivers of these switching patterns. Because of a lack of federal protection guarantees that can result in underwriting, beneficiaries who leave MA for FFS are often unable to obtain supplemental (e.g., Medigap) coverage that reduces cost‐sharing.^17^, ^18^ Similarly, beneficiaries with employer‐ or union‐sponsored FFS coverage may lose cost‐sharing support after switching to MA, which could restrict switching for lower‐need beneficiaries. However, we were unable to examine Medigap coverage or actual out‐of‐pocket costs, given limitations with our data; when we estimated a model controlling for states with community rating and guaranteed issue, results were effectively unchanged (Figure S5).^18^ Importantly, we were unable to examine multiple switching for beneficiaries initially enrolled in MA given data limitations; specifically, we were unable to accurately identify MA enrollees with ADRD using the 20% Medicare FFS files. We also chose to exclude dual eligibles from our main analyses, given their ability to more frequently switch enrollment status compared to other beneficiaries. This potentially limits inferences about the ADRD population, however, given that one‐third of dual eligibles have a dementia diagnosis and that one‐third of those with dementia are dual eligibles.^22^, ^23^ However, the magnitudes of associations (between prior MA enrollment and likelihood of future MA enrollment for those with versus without ADRD) were directionally similar across models, if slightly larger in models restricted to those who were not versus those who were dually eligible, suggesting that the “true,” overall strength of association lies somewhere in the middle of those estimates. While consumers' decisions to exit or enroll are strong evidence of preferences including satisfaction levels with care quality, we were unable to enumerate actual reasons for beneficiary switching. Future work should exploit linked claims and survey data, such as the Medicare Current Beneficiary Survey, to identify factors for these multiple switching enrollments. We include beneficiaries who moved (7.0%) during follow‐up, as in prior studies,^4^, ^6^, ^24^ which could change the underlying pool of alternative MA plans available to consumers. Finally, we used the Medicare enrollment database measure originating from Social Security Administration records, which is known to undercount Hispanic and Asian/Pacific Islander populations and is therefore discouraged for use in studies focused on disparities involving them.^25^ For comparison purposes, we present descriptives of our sample using RTI's race measure (Table S7).

These limitations notwithstanding, the findings have implications for Medicare policy. On the one hand, the results may indicate spillovers in costs and care burdens for FFS, due to MA exits for higher‐need patients who then later re‐enroll when needs are lower, or because MA is able to attract lower‐cost individuals within higher‐cost populations.^21^ If so, the Centers for Medicare and Medicaid Services (CMS) might explore whether MA's care management, including the quality of post‐acute care referrals, is less than satisfactory to patients who re‐enroll in MA but who re‐enroll due to lower premiums—therefore accepting less than adequate care quality.

Alternatively, common re‐entry into MA from FFS, including among high‐need Medicare beneficiaries, may suggest less long‐lasting dissatisfaction with MA and potentially more dissatisfaction with FFS. Legislative or regulatory steps to address cost concerns (related to inability to purchase Medigap supplemental coverage for some beneficiaries) might reduce multiple switching, given its potentially harmful consequences for patient care continuity and patient satisfaction. Moreover, adjusting to new plan coverage rules, provider networks, and providers, while managing health conditions may be additional costs faced by consumers, family members, and non‐family caregivers.^26^

A final potential implication is the complexity of navigating the commercial insurance market. Common switching within MA and across MA and FFS may reflect this complexity and limited use of educational materials for plan comparisons shopping such as the Medicare Plan Finder that offer guidance about expected out‐of‐pocket costs and plan quality.^27^ Absent greater use of these tools and efforts to constrain favorable selection into MA, MA coverage is likely to continue to appeal to consumers in the long term. This may be due to preferences for lower premiums,^14^ despite actual care experiences and MA exits that are suggestive of consumer dissatisfaction, because of MA's use of data on current and prior enrollees to identify, attract, and retain more profitable patients, or due to changes to MA such as broader enrollment criteria and increasingly consumer‐friendly benefits involving long‐term services and supports.^28^

### 4.2 Conclusion

Older Medicare beneficiaries who leave MA for FFS are much likelier to re‐enroll in MA than those who had no prior MA enrollment. That MA remains popular even among those with prior MA disenrollment suggests less dissatisfaction with MA than previously thought, or acceptance of lower‐cost but lower‐quality care, or a failure to educate patients as to the relative costs and benefits of insurance coverage options. Absent policy changes, FFS may struggle to remain relevant in consumers' insurance choices, while potentially bearing the financial brunt of consumers' decisions to use FFS when their needs and costs are relatively high.

## FUNDING INFORMATION

Research reported herein was supported by a grant from the NIA (1R01AG074944‐01). The content is solely the responsibility of the authors and does not necessarily represent the official views of the NIA.

## Supporting information


Data S1.
