# Supplementary material for: Should I stay or should I go again: Multiple switching between fee‐for‐service Medicare and Medicare advantage among older beneficiaries
Source: Health Serv Res. 2024 Oct 17;60(Suppl 2):e14398. doi: 10.1111/1475-6773.14398 (PMC12047697; doi:10.1111/1475-6773.14398)

**Appendix**

**eFigure1.** Illustration of baseline fee-for-service (FFS) and Medicare Advantage (MA) lookback and follow-up periods.

**eTable 1**. Sample derivation and variable missingness

**eTable 2.** Patient, county-level provider, and Medicare Advantage (MA) characteristics, overall and by prior MA enrollment

**eFigure 2**. Sankey chart showing changes in plan type for beneficiaries re-enrolling in MA

**eFigure 3**. Sankey chart showing changes in plan star rating for beneficiaries re-enrolling in MA

**eTable 3**. Cox proportional hazards model result for time to switch to MA

**eTable 4**. Cox proportional hazards model result for time to switch to MA, among beneficiaries with more than one chronic condition during baseline year

**eTable 5**. Cox proportional hazards model result for time to switch to MA, among beneficiaries with nursing home admissions during baseline year

**eTable 6**. Cox proportional hazards model result for time to switch to MA, among dual eligible beneficiaries

**eTable 7**. RTI race variable

**eFigure 4**. Difference in switching to MA conditional on baseline FFS, by prior MA enrollment and MA status, among beneficiaries in areas with high and low MA plan availability

**eFigure 5**. Differences in switching, by ADRD status, controlling for states’ community rating and guaranteed issue.

eFigure 1. Illustration of Baseline Fee-for-service (FFS) and Medicare Advantage (MA) lookback and follow-up periods.


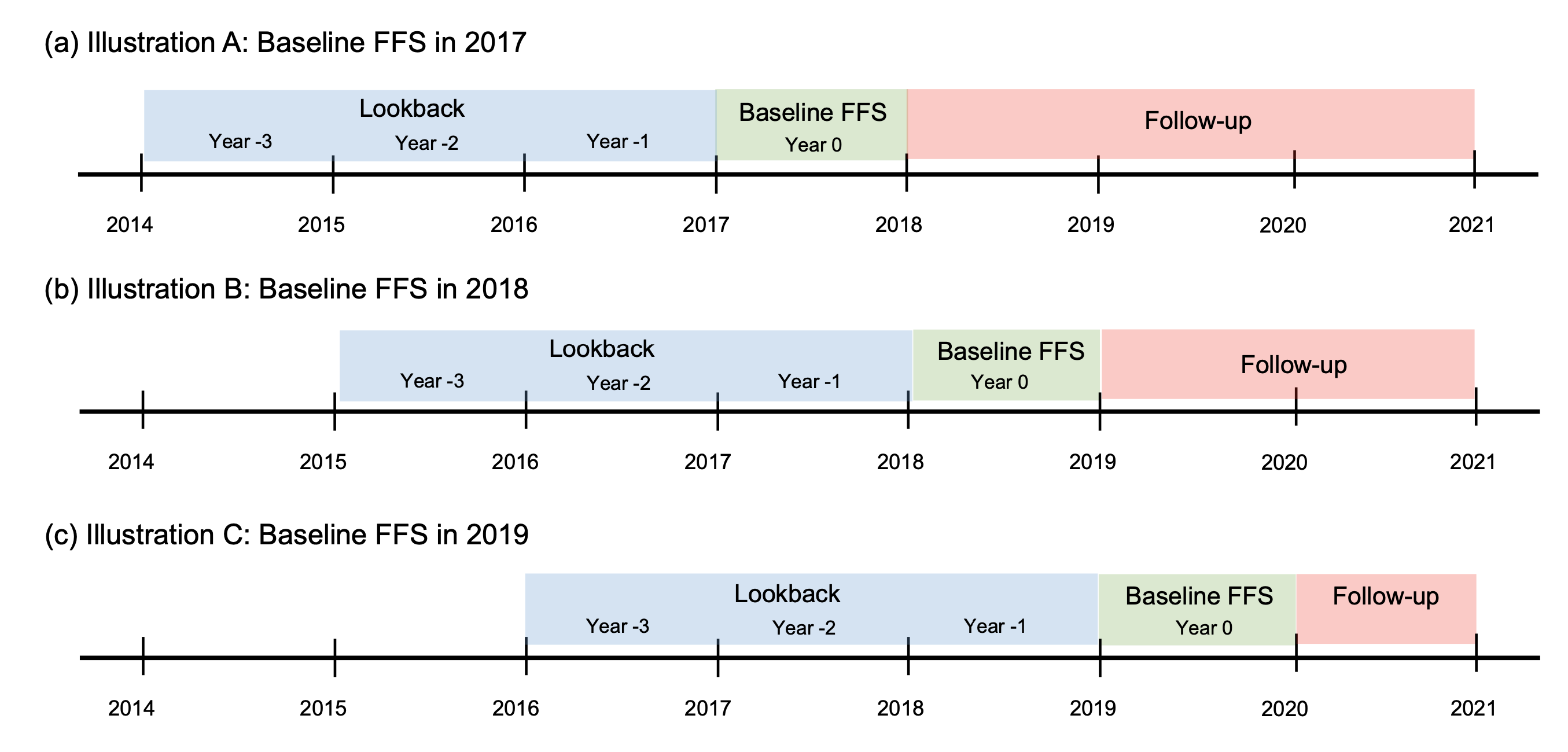


**eTable 1**. Sample derivation and variable missingness

| *Cohort Derivation* | | | | | | | |
| --- | --- | --- | --- | --- | --- | --- | --- |
|  |  | No. observations |  | No. unique beneficiaries |  |  | |
| All non-dual eligible population |  | 11,270,911 |  | 4,084,056 |  |  | |
| Drop non-U.S. counties, missing ZIPs |  | 11,016,947 |  | 3,993,024 |  |  | |
| Drop missing covariates |  | 10,738,112 |  | 3,905,415 |  |  | |
|  |  |  |  |  |  |  | |
| *Variable Missingness* | | | | | | |  |
| Variable |  | No. observations |  | No. missing |  | Percent missing | |
| MA enrollment 1 year prior |  | 11,016,947 |  | 0 |  | 0.0% | |
| MA enrollment 2 years prior |  | 11,016,947 |  | 0 |  | 0.0% | |
| MA enrollment 3 years prior |  | 11,016,947 |  | 0 |  | 0.0% | |
| ADRD |  | 11,016,947 |  | 0 |  | 0.0% | |
| Age at baseline |  | 11,016,947 |  | 0 |  | 0.0% | |
| Female |  | 11,016,947 |  | 0 |  | 0.0% | |
| Race |  | 10,868,471 |  | 148,476 |  | 1.3% | |
| No. inpatient stays |  | 11,016,876 |  | 71 |  | 0.0% | |
| No. SNF admissions |  | 11,016,876 |  | 71 |  | 0.0% | |
| No. HHA visits |  | 11,016,876 |  | 71 |  | 0.0% | |
| No. comorbidities |  | 11,016,947 |  | 0 |  | 0.0% | |
| Median HH income |  | 10,975,014 |  | 41,933 |  | 0.4% | |
| Percent black residents |  | 10,998,393 |  | 18,554 |  | 0.2% | |
| Percent Hispanic residents |  | 10,998,393 |  | 18,554 |  | 0.2% | |
| Percent Asian residents |  | 10,998,393 |  | 18,554 |  | 0.2% | |
| Hospital bed count |  | 11,011,074 |  | 5,873 |  | 0.1% | |
| Percent teaching hospitals |  | 10,780,662 |  | 236,285 |  | 2.1% | |
| No. specialists/100,000 |  | 11,003,861 |  | 13,086 |  | 0.1% | |
| No. PCP/100,000 |  | 11,003,861 |  | 13,086 |  | 0.1% | |
| No. MA plans |  | 11,007,266 |  | 9,681 |  | 0.1% | |
| MA penetration rate |  | 10,991,142 |  | 25,805 |  | 0.2% | |

**eTable 2**. Patient, County-Level Provider, and Medicare Advantage (MA) Characteristics, Overall and by Prior MA Enrollment

|  |  |  |  | Prior MA enrollment | | |
| --- | --- | --- | --- | --- | --- | --- |
|  |  | Overall |  | No |  | Yes |
| *Patients* |  | N=3,993,024 |  | N=3,846,945 |  | N=146,079 |
| Age |  | 76 (7.1) |  | 76.0 (7.1) |  | 75.5 (6.5) |
| Female (%) |  | 53.9 |  | 53.9 |  | 55.1 |
| Race (%) |  |  |  |  |  |  |
| Black |  | 1.3 |  | 1.3 |  | 1.1 |
| White |  | 88.4 |  | 88.5 |  | 86.7 |
| Asian |  | 6.1 |  | 6.1 |  | 7.7 |
| Hispanic |  | 1.8 |  | 1.8 |  | 2.0 |
| Other |  | 1.2 |  | 1.2 |  | 1.5 |
| North American Native |  | 0.7 |  | 0.7 |  | 0.8 |
| Unknown |  | 0.4 |  | 0.4 |  | 0.3 |
| ADRD (%) |  | 7.8 |  | 7.8 |  | 8.2 |
| No. inpatient stays |  | 0.2 (0.7) |  | 0.2 (0.7) |  | 0.3 (0.7) |
| No. SNF admissions |  | 0.0 (0.3) |  | 0.0 (0.3) |  | 0.1 (0.3) |
| No. HHA visits |  | 0.1 (0.6) |  | 0.1 (0.6) |  | 0.2 (0.7) |
| No. comorbidities |  | 1.0 (1.5) |  | 1.0 (1.5) |  | 1.1 (1.6) |
| *Provider and MA* |  |  |  |  |  |  |
| Hospital bed count |  | 2,385.7 (4,143.5) |  | 2,381.5 (4,139.6) |  | 2,495.6 (4,243.3) |
| Median HH income |  | 65,453.6 (26,975.4) |  | 65,538.2 (27,037.4) |  | 63,228.6 (25,187.7) |
| Black residents (%) |  | 9.9 (15.6) |  | 10.0 (15.5) |  | 9.6 (15.8) |
| Hispanic residents (%) |  | 12.0 (15.0) |  | 12.0 (15.0) |  | 12.0 (15.4) |
| Asian residents (%) |  | 4.4 (7.3) |  | 4.4 (7.3) |  | 4.2 (7.5) |
| Teaching hospitals (%) |  | 5.7 (10.0) |  | 5.7 (10.1) |  | 5.7 (9.6) |
| No. MA plans |  | 39.0 (17.7) |  | 38.9 (17.7) |  | 40.0 (18.1) |
| MA penetration rate (%) |  | 30.4 (13.7) |  | 30.3 (13.7) |  | 32.8 (13.5) |
| No. specialists/100,000 |  | 97.7 (82.1) |  | 97.8 (82.3) |  | 95.6 (76.8) |
| No. PCP/100,000 |  | 75.0 (32.7) |  | 75.1 (31.8) |  | 73.9 (30.3) |

*Note*: Cells show either percentage or mean (SD). MA = Medicare Advantage; ADRD = Alzheimer’s Disease and Related Dementias; SNF=skilled nursing facility; HHA= home health agency; HH = household; PCP = primary care provider.

**eFigure 2**. Sankey chart showing changes in plan type for beneficiaries re-enrolling in MA


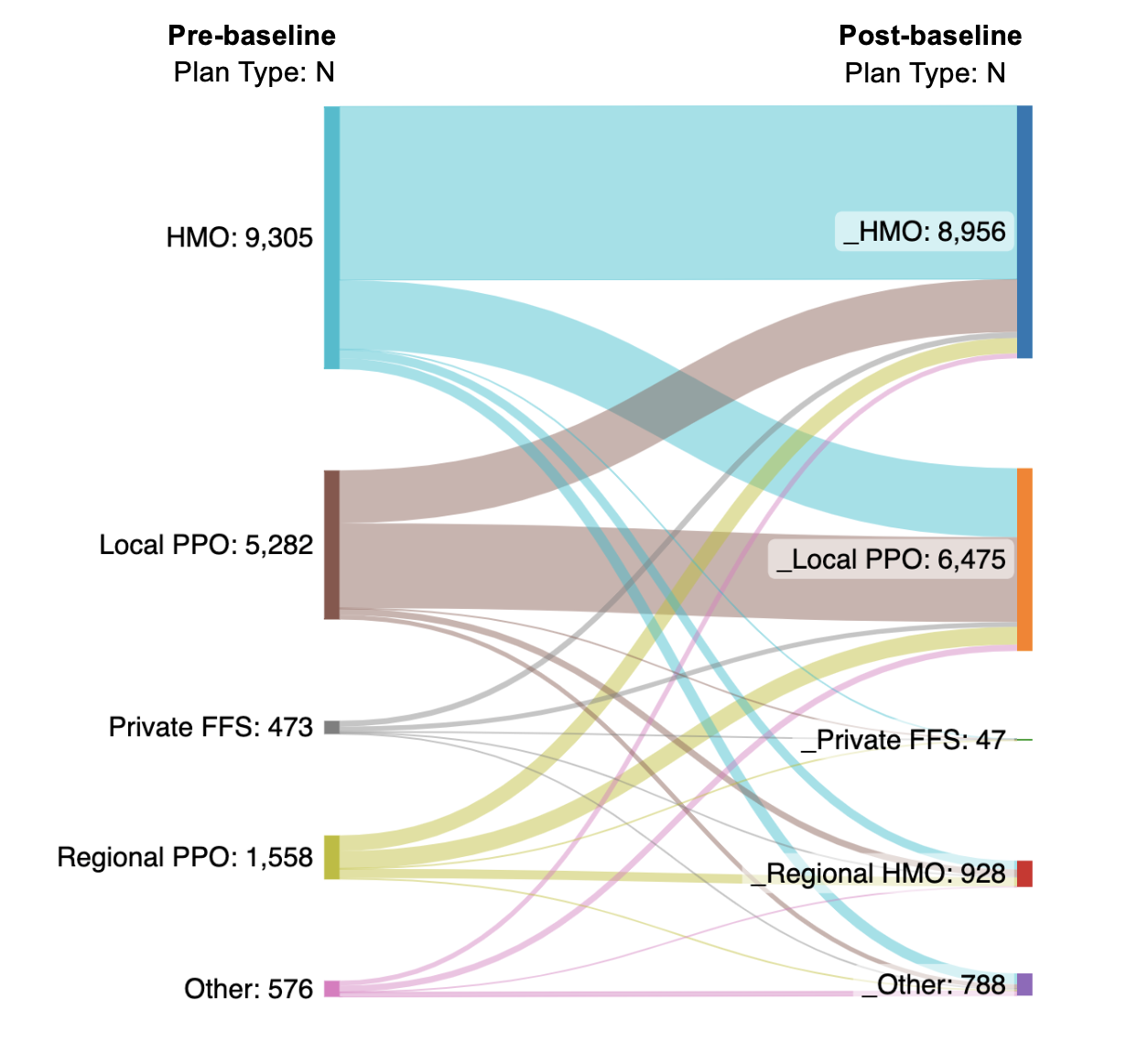


*Note*: Data on the left side of the chart illustrate plan type and enrollment volume during prior MA enrollment (i.e., prior to the baseline FFS enrollment) while data on the right side of the chart illustrate plan type and enrollment volume during subsequent MA enrollment (i.e., after the baseline FFS enrollment). For instance, there were 9,305 MA enrollees in HMO plans in the period prior to baseline compared to 8,956 in HMO plans in the period after baseline. The lines illustrate increases or decreases in enrollment volume, by plan star rating, over time (i.e., from before to after baseline).

**eFigure 3**. Sankey chart showing changes in plan star rating for beneficiaries re-enrolling in MA


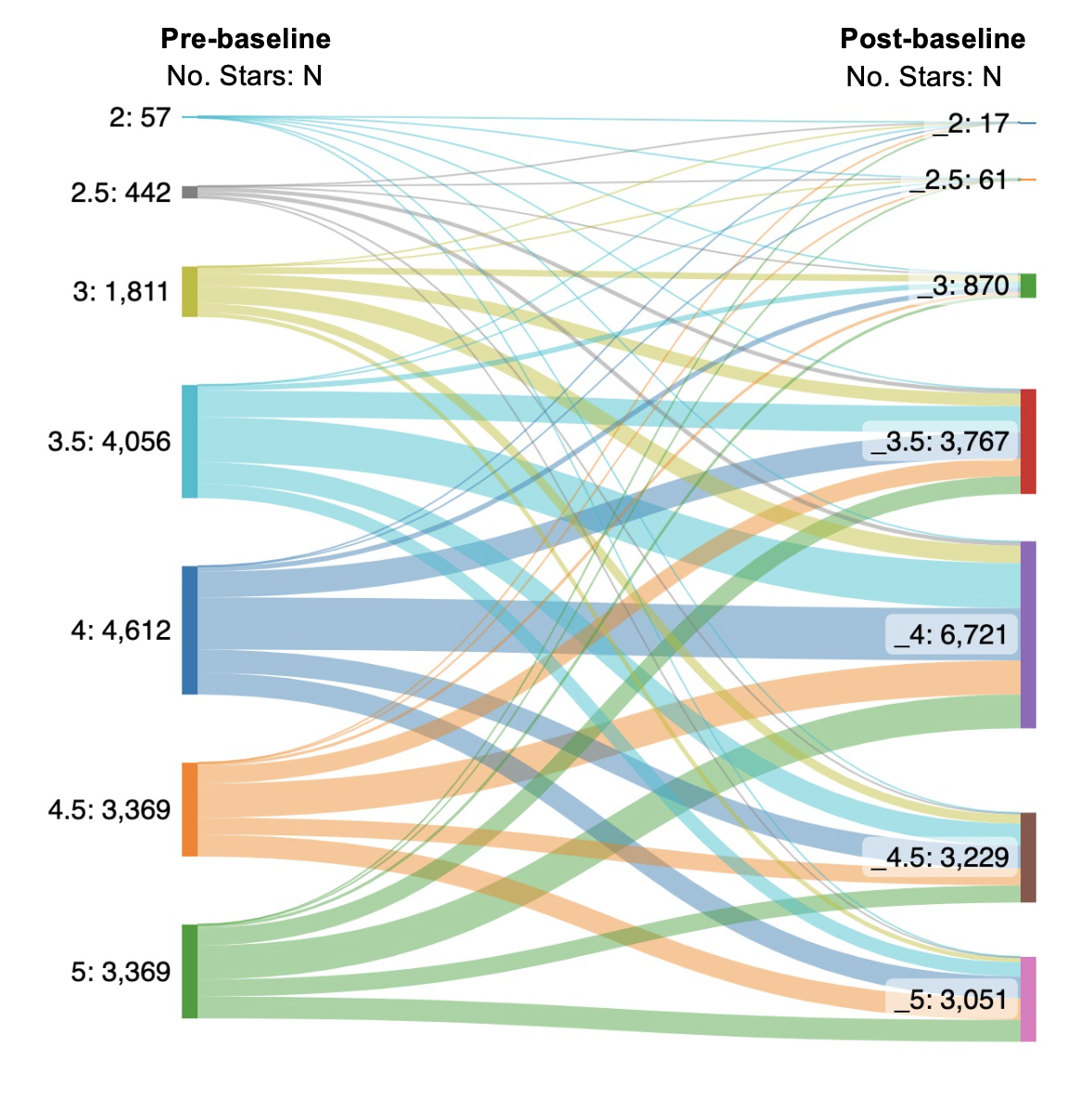


*Note*: Data on the left side of the chart illustrate plan star ratings and enrollment volume during prior MA enrollment (i.e., prior to the baseline FFS enrollment) while data on the right side of the chart illustrate plan star ratings and enrollment volume during subsequent MA enrollment (i.e., after the baseline FFS enrollment). For instance, there were 3,369 MA enrollees in plans with 5 stars in the period prior to baseline compared to 3,051 in plans with 5 stars in the period after baseline. The lines illustrate increases or decreases in enrollment volume, by plan star rating, over time (i.e., from before to after baseline).

**eTable 3**. Cox Proportional Hazards Model Result for Time to Switch to MA

|  |  | Coefficient |  | Std. Err. |  | *P value* |  | Hazard Ratio |
| --- | --- | --- | --- | --- | --- | --- | --- | --- |
| MA enrollment 1 year prior |  | 0.98 |  | 0.01 |  | <.0001 |  | 2.65 |
| MA enrollment 2 years prior |  | 0.26 |  | 0.01 |  | <.0001 |  | 1.30 |
| MA enrollment 3 years prior |  | 0.63 |  | 0.01 |  | <.0001 |  | 1.89 |
| ADRD |  | 0.05 |  | 0.01 |  | <.0001 |  | 1.05 |
| MA enrollment 1 year prior * ADRD |  | -0.04 |  | 0.04 |  | 0.3286 |  | 0.96 |
| MA enrollment 2 years prior * ADRD |  | -0.03 |  | 0.05 |  | 0.5909 |  | 0.98 |
| MA enrollment 3 years prior * ADRD |  | 0.26 |  | 0.03 |  | <.0001 |  | 1.30 |
| Age at baseline |  | -0.03 |  | 0.00 |  | <.0001 |  | 0.97 |
| Female |  | -0.01 |  | 0.00 |  | 0.0046 |  | 0.99 |
| Race |  |  |  |  |  |  |  |  |
| Black |  | 0.50 |  | 0.01 |  | <.0001 |  | 1.65 |
| Asian |  | 0.32 |  | 0.01 |  | <.0001 |  | 1.38 |
| Hispanic |  | 0.28 |  | 0.02 |  | <.0001 |  | 1.32 |
| Other |  | 0.10 |  | 0.01 |  | <.0001 |  | 1.10 |
| North American Native |  | -0.22 |  | 0.04 |  | <.0001 |  | 0.80 |
| Unknown Race |  | -0.04 |  | 0.01 |  | 0.003 |  | 0.96 |
| No. inpatient stays |  | -0.08 |  | 0.00 |  | <.0001 |  | 0.92 |
| No. SNF admissions |  | 0.00 |  | 0.01 |  | 0.8737 |  | 1.00 |
| No. HHA visits |  | 0.03 |  | 0.00 |  | <.0001 |  | 1.03 |
| No. comorbidities |  | -0.02 |  | 0.00 |  | <.0001 |  | 0.98 |
| Provider and MA plan |  |  |  |  |  |  |  |  |
| Hospital bed count |  | 0.00 |  | 0.00 |  | <.0001 |  | 1.00 |
| Median HH income |  | 0.00 |  | 0.00 |  | <.0001 |  | 1.00 |
| Percent black residents |  | 0.00 |  | 0.00 |  | <.0001 |  | 1.00 |
| Percent Hispanic residents |  | 0.00 |  | 0.00 |  | <.0001 |  | 1.00 |
| Percent Asian residents |  | 0.00 |  | 0.00 |  | 0.0008 |  | 1.00 |
| Percent teaching hospitals |  | 0.00 |  | 0.00 |  | 0.0443 |  | 1.00 |
| No. specialists/100,000 |  | 0.00 |  | 0.00 |  | <.0001 |  | 1.00 |
| No. PCP/100,000 |  | 0.00 |  | 0.00 |  | 0.0004 |  | 1.00 |
| No. MA plans |  | 0.01 |  | 0.00 |  | <.0001 |  | 1.01 |
| MA penetration rate |  | 0.03 |  | 0.00 |  | <.0001 |  | 1.03 |

*Note*: MA = Medicare Advantage; ADRD = Alzheimer’s Disease and Related Dementias; No. = number; SNF=skilled nursing facility; HHA= home health agency; HH = household; PCP = primary care provider.

**eTable 4**. Cox Proportional Hazards Model Result for Time to Switch to MA, among Beneficiaries with More than One Chronic Condition during Baseline Year

|  |  | Coefficient |  | Std. Err. |  | P value |  | Hazard Ratio |
| --- | --- | --- | --- | --- | --- | --- | --- | --- |
| MA enrollment 1 year prior |  | 0.92 |  | 0.02 |  | <.0001 |  | 2.50 |
| MA enrollment 2 years prior |  | 0.16 |  | 0.02 |  | <.0001 |  | 1.18 |
| MA enrollment 3 years prior |  | 0.66 |  | 0.02 |  | <.0001 |  | 1.93 |
| ADRD |  | 0.03 |  | 0.01 |  | <.0001 |  | 1.04 |
| MA enrollment 1 year prior* ADRD |  | -0.05 |  | 0.05 |  | 0.3783 |  | 0.96 |
| MA enrollment 2 years prior* ADRD |  | 0.03 |  | 0.06 |  | 0.6316 |  | 1.03 |
| MA enrollment 3 years prior* ADRD |  | 0.20 |  | 0.04 |  | <.0001 |  | 1.22 |
| Age at baseline |  | -0.02 |  | 0.00 |  | <.0001 |  | 0.98 |
| Female |  | -0.06 |  | 0.01 |  | <.0001 |  | 0.94 |
| Race |  |  |  |  |  |  |  |  |
| Black |  | 0.58 |  | 0.01 |  | <.0001 |  | 1.78 |
| Asian |  | 0.25 |  | 0.03 |  | <.0001 |  | 1.29 |
| Hispanic |  | 0.60 |  | 0.03 |  | <.0001 |  | 1.82 |
| Other |  | 0.04 |  | 0.02 |  | 0.0755 |  | 1.04 |
| North American Native |  | -0.17 |  | 0.05 |  | 0.0011 |  | 0.85 |
| Unknown Race |  | -0.11 |  | 0.03 |  | 0.0002 |  | 0.89 |
| No. inpatient stays |  | -0.07 |  | 0.00 |  | <.0001 |  | 0.93 |
| No. SNF admissions |  | -0.01 |  | 0.01 |  | 0.4095 |  | 0.99 |
| No. HHA visits |  | 0.03 |  | 0.00 |  | <.0001 |  | 1.03 |
| No. comorbidities |  | -0.03 |  | 0.00 |  | <.0001 |  | 0.97 |
| Provider and MA plan |  |  |  |  |  |  |  |  |
| Hospital bed count |  | 0.00 |  | 0.00 |  | <.0001 |  | 1.00 |
| Median HH income |  | 0.00 |  | 0.00 |  | <.0001 |  | 1.00 |
| Percent black residents |  | 0.00 |  | 0.00 |  | 0.0004 |  | 1.00 |
| Percent Hispanic residents |  | 0.00 |  | 0.00 |  | <.0001 |  | 1.00 |
| Percent Asian residents |  | 0.00 |  | 0.00 |  | <.0001 |  | 1.00 |
| Percent teaching hospitals |  | 0.00 |  | 0.00 |  | 0.0034 |  | 1.00 |
| No. specialists/100,000 |  | 0.00 |  | 0.00 |  | 0.065 |  | 1.00 |
| No. PCP/100,000 |  | 0.00 |  | 0.00 |  | 0.0275 |  | 1.00 |
| No. MA plans |  | 0.01 |  | 0.00 |  | <.0001 |  | 1.01 |
| MA penetration rate |  | 0.03 |  | 0.00 |  | <.0001 |  | 1.03 |

*Note*: MA = Medicare Advantage; ADRD = Alzheimer’s Disease and Related Dementias; No. = number. SNF=skilled nursing facility; HHA= home health agency; HH = household; PCP = primary care provider.

**eTable 5**. Cox Proportional Hazards Model Result for Time to Switch to MA, among Beneficiaries with Nursing Home Admissions during Baseline Year

|  |  | Coefficient |  | Std. Err. |  | P value |  | Hazard Ratio |
| --- | --- | --- | --- | --- | --- | --- | --- | --- |
| MA enrollment 1 year prior |  | 1.08 |  | 0.08 |  | <.0001 |  | 2.95 |
| MA enrollment 2 years prior |  | 0.06 |  | 0.10 |  | 0.5364 |  | 1.07 |
| MA enrollment 3 years prior |  | 0.79 |  | 0.07 |  | <.0001 |  | 2.20 |
| ADRD |  | 0.06 |  | 0.02 |  | 0.0168 |  | 1.06 |
| MA enrollment 1 year prior* ADRD |  | -0.08 |  | 0.13 |  | 0.561 |  | 0.93 |
| MA enrollment 2 years prior* ADRD |  | 0.08 |  | 0.17 |  | 0.6564 |  | 1.08 |
| MA enrollment 3 years prior* ADRD |  | 0.26 |  | 0.11 |  | 0.0218 |  | 1.30 |
| Age at baseline |  | -0.02 |  | 0.00 |  | <.0001 |  | 0.98 |
| Female |  | -0.05 |  | 0.02 |  | 0.04 |  | 0.96 |
| Race |  |  |  |  |  |  |  |  |
| Black |  | 0.61 |  | 0.04 |  | <.0001 |  | 1.84 |
| Asian |  | 0.23 |  | 0.13 |  | 0.0881 |  | 1.26 |
| Hispanic |  | 0.38 |  | 0.15 |  | 0.0114 |  | 1.46 |
| Other |  | -0.10 |  | 0.12 |  | 0.4165 |  | 0.91 |
| North American Native |  | -0.27 |  | 0.25 |  | 0.2657 |  | 0.76 |
| Unknown Race |  | -0.21 |  | 0.18 |  | 0.2533 |  | 0.81 |
| No. inpatient stays |  | -0.07 |  | 0.01 |  | <.0001 |  | 0.93 |
| No. SNF admissions |  | 0.00 |  | 0.02 |  | 0.806 |  | 1.00 |
| No. HHA visits |  | 0.05 |  | 0.01 |  | <.0001 |  | 1.05 |
| No. comorbidities |  | -0.01 |  | 0.01 |  | 0.0245 |  | 0.99 |
| Provider and MA plan |  |  |  |  |  |  |  |  |
| Hospital bed count |  | 0.00 |  | 0.00 |  | 0.8841 |  | 1.00 |
| Median HH income |  | 0.00 |  | 0.00 |  | <.0001 |  | 1.00 |
| Percent black residents |  | 0.00 |  | 0.00 |  | 0.3461 |  | 1.00 |
| Percent Hispanic residents |  | 0.00 |  | 0.00 |  | 0.0483 |  | 1.00 |
| Percent Asian residents |  | 0.00 |  | 0.00 |  | 0.9219 |  | 1.00 |
| Percent teaching hospitals |  | 0.00 |  | 0.01 |  | 0.9469 |  | 1.00 |
| No. specialists/100,000 |  | 0.00 |  | 0.00 |  | 0.0508 |  | 1.00 |
| No. PCP/100,000 |  | 0.00 |  | 0.00 |  | 0.4899 |  | 1.00 |
| No. MA plan |  | 0.00 |  | 0.00 |  | 0.6433 |  | 1.00 |
| MA penetration rate |  | 0.03 |  | 0.00 |  | <.0001 |  | 1.03 |

*Note*: MA = Medicare Advantage; ADRD = Alzheimer’s Disease and Related Dementias; SNF=skilled nursing facility; HHA= home health agency; HH = household; PCP = primary care provider.

**eTable 6**. Cox Proportional Hazards Model Result for Time to Switch to MA, among Dual Eligible Beneficiaries

|  |  | Coefficient |  | Std. Err. |  | P value |  | Hazard Ratio | |
| --- | --- | --- | --- | --- | --- | --- | --- | --- | --- |
| MA enrollment 1 year prior |  | 0.88 |  | 0.02 |  | <.0001 |  | 2.41 |  |
| MA enrollment 2 years prior |  | 0.55 |  | 0.02 |  | <.0001 |  | 1.74 |  |
| MA enrollment 3 years prior |  | 0.48 |  | 0.02 |  | <.0001 |  | 1.61 |  |
| ADRD |  | -0.11 |  | 0.01 |  | <.0001 |  | 0.90 |  |
| MA enrollment 1 year prior * ADRD |  | -0.33 |  | 0.03 |  | <.0001 |  | 0.72 |  |
| MA enrollment 2 years prior * ADRD |  | -0.23 |  | 0.04 |  | <.0001 |  | 0.80 |  |
| MA enrollment 3 years prior * ADRD |  | -0.16 |  | 0.04 |  | <.0001 |  | 0.85 |  |
| Age at baseline |  | -0.03 |  | 0.00 |  | <.0001 |  | 0.97 |  |
| Female |  | -0.01 |  | 0.01 |  | 0.0579 |  | 0.99 |  |
| Race |  |  |  |  |  |  |  |  |  |
| Black |  | 0.28 |  | 0.01 |  | <.0001 |  | 1.32 |  |
| Asian |  | 0.03 |  | 0.01 |  | 0.0332 |  | 1.03 |  |
| Hispanic |  | 0.17 |  | 0.01 |  | <.0001 |  | 1.18 |  |
| Other |  | 0.04 |  | 0.02 |  | 0.0317 |  | 1.05 |  |
| North American Native |  | -0.24 |  | 0.05 |  | <.0001 |  | 0.79 |  |
| Unknown Race |  | 0.36 |  | 0.02 |  | <.0001 |  | 1.44 |  |
| No. inpatient stays |  | -0.07 |  | 0.01 |  | <.0001 |  | 0.94 |  |
| No. SNF admissions |  | 0.04 |  | 0.01 |  | <.0001 |  | 1.05 |  |
| No. HHA visits |  | -0.05 |  | 0.00 |  | <.0001 |  | 0.95 |  |
| No. comorbidities |  | -0.05 |  | 0.00 |  | <.0001 |  | 0.95 |  |
| Provider and MA plan |  |  |  |  |  |  |  |  |  |
| Hospital bed count |  | 0.00 |  | 0.00 |  | <.0001 |  | 1.00 |  |
| Median HH income |  | 0.00 |  | 0.00 |  | <.0001 |  | 1.00 |  |
| Percent black residents |  | 0.00 |  | 0.00 |  | <.0001 |  | 1.00 |  |
| Percent Hispanic residents |  | 0.01 |  | 0.00 |  | <.0001 |  | 1.01 |  |
| Percent Asian residents |  | 0.00 |  | 0.00 |  | <.0001 |  | 1.00 |  |
| Percent teaching hospitals |  | 0.01 |  | 0.00 |  | 0.0005 |  | 1.01 |  |
| No. specialists/100,000 |  | 0.00 |  | 0.00 |  | 0.2976 |  | 1.00 |  |
| No. PCP/100,000 |  | 0.00 |  | 0.00 |  | 0.3474 |  | 1.00 |  |
| No. MA plans |  | 0.02 |  | 0.00 |  | <.0001 |  | 1.02 |  |
| MA penetration rate |  | 0.03 |  | 0.00 |  | <.0001 |  | 1.03 |  |

*Note*: MA = Medicare Advantage; ADRD = Alzheimer’s Disease and Related Dementias; No. = number. SNF=skilled nursing facility; HHA= home health agency; HH = household; PCP = primary care provider.

**eTable 7**. RTI race variable

|  |  |  |  | Prior MA enrollment | | |
| --- | --- | --- | --- | --- | --- | --- |
|  |  | Overall |  | No |  | Yes |
| Patients (N) |  | 3,993,024 |  | 3,846,945 |  | 146,079 |
| RTI race (%) |  |  |  |  |  |  |
| Unknown |  | 1.2 |  | 1.3 |  | 1.0 |
| Non-Hispanic White |  | 85.7 |  | 85.8 |  | 83.4 |
| Black (or African-American) |  | 6.1 |  | 6.0 |  | 7.6 |
| Other |  | 0.9 |  | 0.9 |  | 0.9 |
| Asian/Pacific Islander |  | 2.1 |  | 2.0 |  | 2.5 |
| Hispanic |  | 3.7 |  | 3.7 |  | 4.3 |
| American Indian/Alaskan Native |  | 0.4 |  | 0.4 |  | 0.3 |

**eFigure 4**. Difference in switching to MA conditional on baseline FFS, by prior MA enrollment and MA status, among beneficiaries in areas with high and low MA plan availability


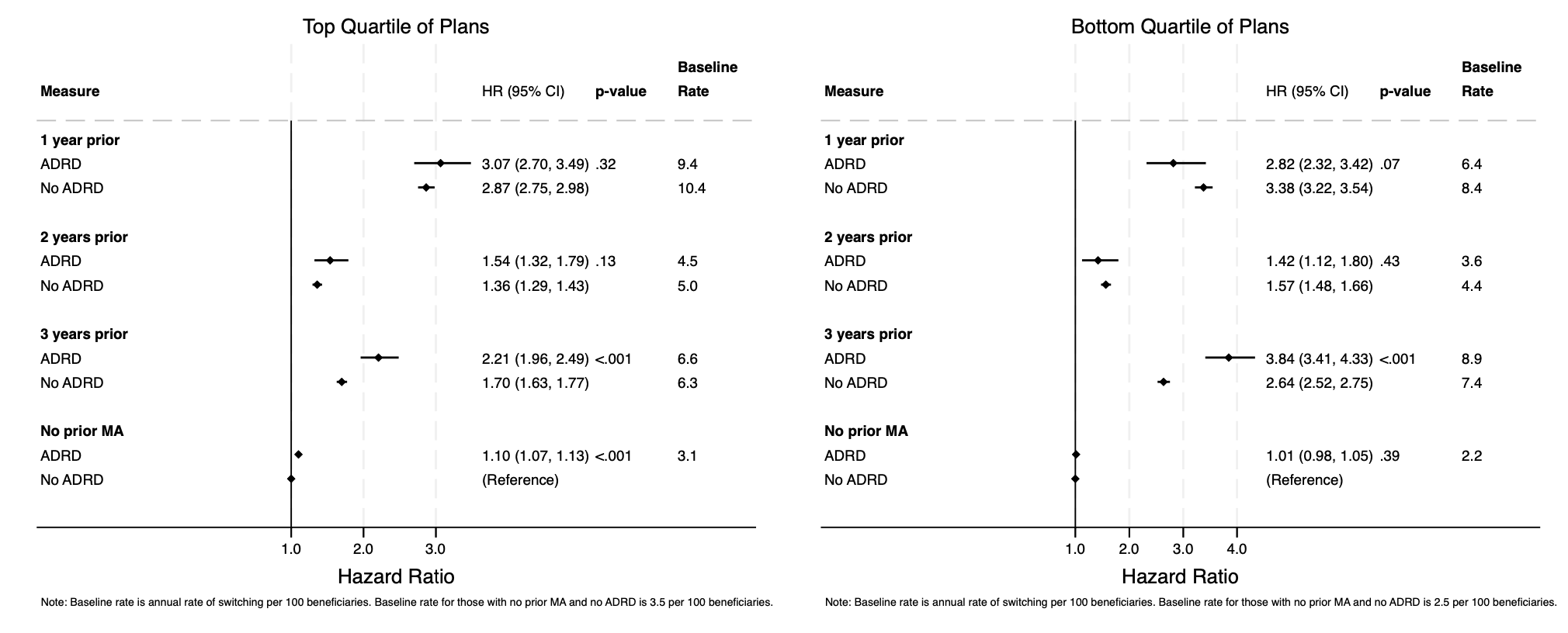


*Note:* Authors’ analysis of data from the Medicare Master Beneficiary Summary File (MBSF) linked to Medicare claims. Provider and MA plan characteristics are from the Agency on Healthcare Research and Quality’s Social Determinants of Health (SDOH) database and Medicare Advantage/Part D Contract and Enrollment Data. Hazard ratios are calculated from the Cox model coefficients. Non-ADRD beneficiaries with no prior MA enrollment is the reference group. The p-values show the significance level comparing ADRD and non ADRD population. *P*-values are for the contrasts between ADRD and non-ADRD populations, i.e., the risk differences in FFS-to-MA switching for those with versus without ADRD among those with MA enrollment 1 year prior compared to those without ADRD who had no prior MA experience.

**eFigure 5**. Differences in switching, by ADRD status, controlling for states’ community rating and guaranteed issue.


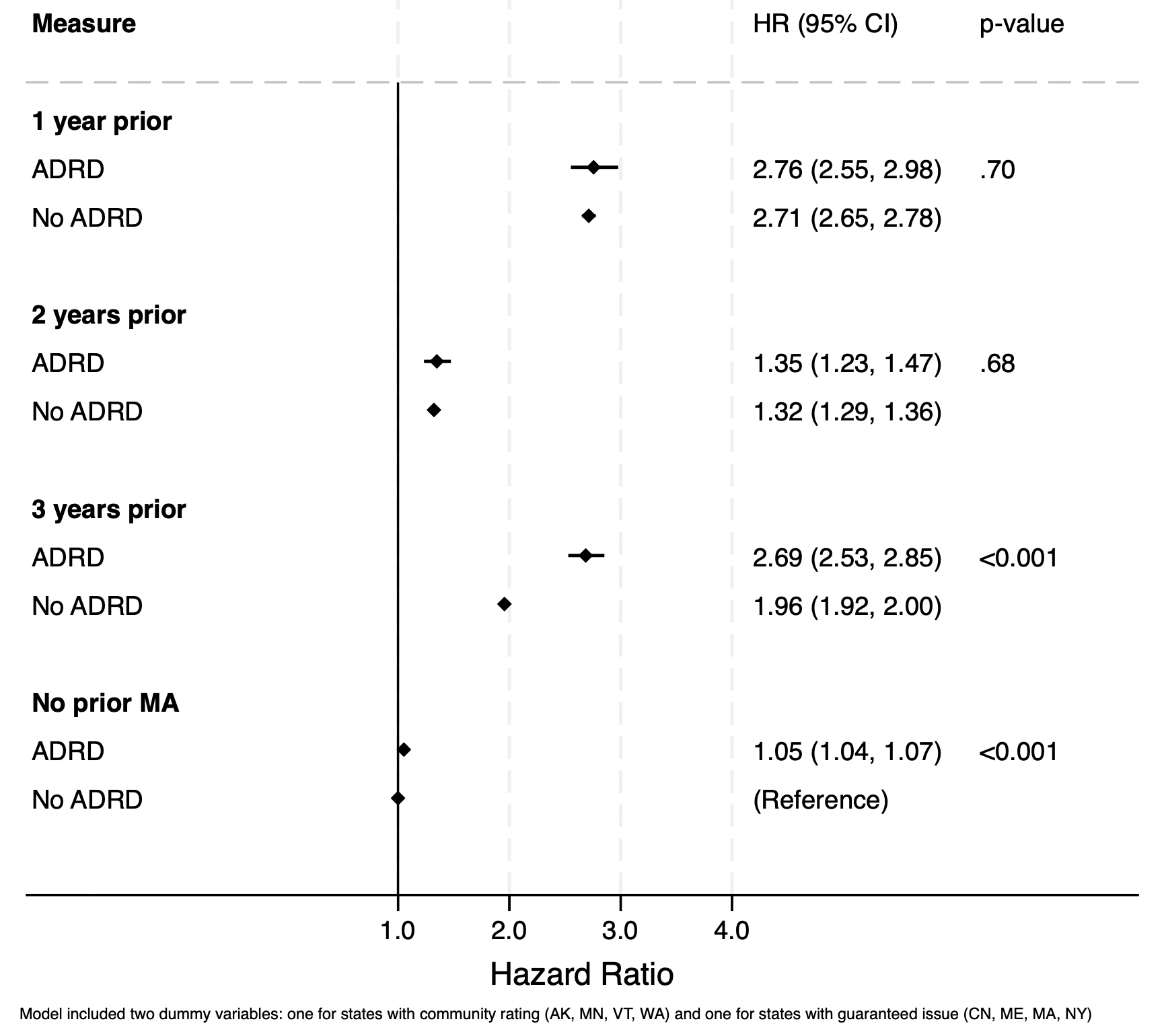

Supplement: Supplementary file 1 — Data S1. [file HESR-60-0-s001.docx]
